# A Contextual Analysis and Logic Model for Integrated Care for Frail Older Adults Living at Home: The INSPIRE Project

**DOI:** 10.5334/ijic.5607

**Published:** 2021-04-23

**Authors:** Olivia Yip, Evelyn Huber, Samuel Stenz, Leah L. Zullig, Andreas Zeller, Sabina M. De Geest, Mieke Deschodt, the INSPIRE consortium

**Affiliations:** 1 Nursing Science (INS), Department of Public Health, University of Basel, Switzerland; 2 Institute of Nursing, School of Health Professions, ZHAW Zurich University of Applied Sciences, CH; 3 Department of Population Health Sciences, Duke University Medical Center, USA; 4 Centre for Primary Health Care, University of Basel, Switzerland; 5 Academic Center for Nursing and Midwifery, Department of Public Health and Primary Care, KU Leuven, Belgium; 6 Gerontology and Geriatrics, Department of Public Health and Primary Care, KU Leuven, Belgium; 7 Healthcare and Ethics, Faculty of Medicine and Life Sciences, UHasselt, Belgium; 8 Matthias Briel, Matthias Schwenkglenks, Franziska Zúñiga, Penelope Vounatsou, Carlos Quinto, Eva Blozik, Flaka Siqeca, Maria José Mendieta Jara

**Keywords:** Integrated care, Implementation science, Frail elderly, Logic model, Program development, Program theory

## Abstract

**Introduction:**

Implementation science methods and a theory-driven approach can enhance the understanding of whether, how, and why integrated care for frail older adults is successful in practice. In this study, we aimed to perform a contextual analysis, develop a logic model, and select preliminary implementation strategies for an integrated care model in newly created information and advice centers for older adults in Canton Basel-Landschaft, Switzerland.

**Methods:**

We conducted a contextual analysis to determine factors which may influence the integrated care model and implementation strategies needed. A logic model depicting the overall program theory, including inputs, core components, outputs and outcomes, was designed using a deductive approach, and included stakeholders’ feedback and preliminary implementation strategies.

**Results:**

Contextual factors were identified (e.g., lack of integrated care regulations, existing community services, and a care pathway needed). Core components of the care model include screening, referral, assessment, care plan creation and coordination, and follow-up. Outcomes included person-centred coordinated care experiences, hospitalization rate and symptom burden, among others. Implementation strategies (e.g., nurse training and co-developing educational materials) were proposed to facilitate care model adoption.

**Conclusion:**

Contextual understanding and a clear logic model should enhance the potential for successful implementation of the integrated care model.

## Introduction

Frail older adults, often living with multimorbidity and functional and cognitive disabilities [1], are at higher risk of mortality, hospitalization and institutionalization [2,3]. Care for this population tends to be uncoordinated and fragmented [4], as frail older adults may require support from several health and social care providers as well as informal care [5,6]. Fragmented care can lead to negative health outcomes such as patient confusion and distress, gaps in information delivery, duplication of services, unnecessary hospitalizations and higher care costs [4]. These negative outcomes may be overcome by integrated care, a person-centered approach where individual pro-active care is facilitated by continuous, multidisciplinary collaboration and coordination of various care providers [7,8].

In the many studies evaluating integrated care for frail or multimorbid older adults, comprehensive assessments, tailored care plans, multidisciplinary care teams, case management, and a proactive and patient-centered approach, are commonly reported as key components [9,10,11,12,13,14,15]. However, systematic reviews indicate major heterogeneity with respect to the target population, the study outcomes selected, the delivery of their intervention elements, and most importantly, the results found on a patient-, provider-, and system-level, impeding consistent conclusions [9,10,11]. The lack of impact resulting from integrated care initiatives may be related to the outcomes measured and the measures used [9,10], but may also be a result of implementation issues with these complex interventions, potentially low fidelity to the intervention or the intervention lacking contextual fit [16,17,18]. This indicated the need for effectiveness studies which include process evaluations, contextual analysis, and measuring implementation outcomes to determine if, how and why community-based integrated care for frail, older adults is successful in practice [14,19,20].

Intervention development, implementation, and evaluation can be facilitated by using a theory-driven approach and implementation science methods, ensuring contextual relevance [21,22]. Furthermore, feasibility studies to measure for example, acceptability and fidelity, are needed before evaluating ultimate effectiveness [22,23,24,25], especially in light of the major challenges recognized in implementing integrated care in practice [17]. While fidelity has been measured in a seldom number of studies of integrated care for frail, home-based older adults [26,27], most studies rarely include implementation science methods such as stakeholder involvement; use of theories, models and frameworks; contextual analysis; and studying implementation strategies (i.e., the methods used to increase the likelihood for intervention uptake and success [28]) and implementation outcomes (e.g., acceptability, adoption, and fidelity [23]) [11,29]. Additionally, logic models, which are recommended when planning an intervention to illustrate how a program will create change [25,30], were not often used [11,18]. Logic models are visual tools that demonstrate an overall program theory, describing and linking the program’s input/resources, activities, expected outcomes and impact [31,32]. They are especially valuable in integrated care initiatives as deciphering the underlying pathway and which individual components of these complex interventions contribute to the outcomes can be especially challenging [11,14]. Logic models have numerous benefits during program planning, monitoring and evaluation, such as communicating the evidence-informed strategies used in the program; detecting gaps in theory; facilitating a shared understanding of the program with stakeholders; identifying what to measure during evaluation; and helping to differentiate between intervention and implementation failure [31,32,33]. Applying implementation science methods and creating a logic model when developing an intervention may improve the chances of success, inform future care models and reduce research waste.

Context is a major focus in implementation science [21,34,35]. During intervention development, a strong grasp of the context helps to ensure that the intervention components will be well-suited for the context and the actions needed [18]. Although there are inconsistencies in how the term “context” is formulated in the literature, Pfadenhauer et al.’s (2017) work using a Pragmatic Utility concept analysis helped to refine the conceptualization of context as: “a set of characteristics and circumstances that consist of active and unique factors, within which the implementation is embedded. As such, context … interacts, influences, modifies and facilitates or constrains the intervention and its implementation” [36]. The Context and Implementation of Complex Interventions (CICI) framework proposed by Pfadenhauer provides a richer assessment of “context”, differentiating it from the “setting” [36]. Specifically, the “context” dimension includes seven domains: geographical, epidemiological, socio-cultural, socio-economic, ethical, legal and political, while “setting” is defined by the physical place where an intervention takes place [36]. As a “determinant” framework, CICI provides a solid basis for understanding and analyzing the extensive set of factors within the context which may affect the intervention and implementation outcomes [36,37,38]. Accounting for such contextual factors is an essential consideration when planning and evaluating integrated care initiatives [7,11,18] and can lead to the selection of appropriate implementation strategies [39,40]. The selection of implementation strategies will be influenced by their proposed effectiveness [41] but also greatly depends on the context in which an intervention is implemented [42].

Given their major importance in the development and evaluation of complex care interventions, implementation science methods and a theory-driven approach will be applied in the INSPIRE project (ImplemeNtation of a community-baSed care Program for home dwelling senIoR citizEns) in Canton Basel-Landschaft (BL), Switzerland. A 2018 Cantonal law required the 86 BL municipalities, with an approximate population of 288’000, to re-organize themselves into eight care regions, and each develop a care concept including services for outpatient, intermediate, and inpatient care [43]. The INSPIRE project aims to develop, implement and evaluate an integrated care model for the information and advice centers (IAC), which are required in each of these newly formed care regions [43]. These community-based centers must include a nurse to provide needs assessments and advice for older adults who are living at home, especially if entry into a nursing home is being considered [43]. Building on gaps and recommendations in recent studies, the aim of this paper is to report the contextual analysis, logic model development, and preliminary implementation strategies for the INSPIRE integrated care model for home-dwelling older adults in Canton Basel-Landschaft.

## Methods

### Overall Project Design

**Figure 1:**
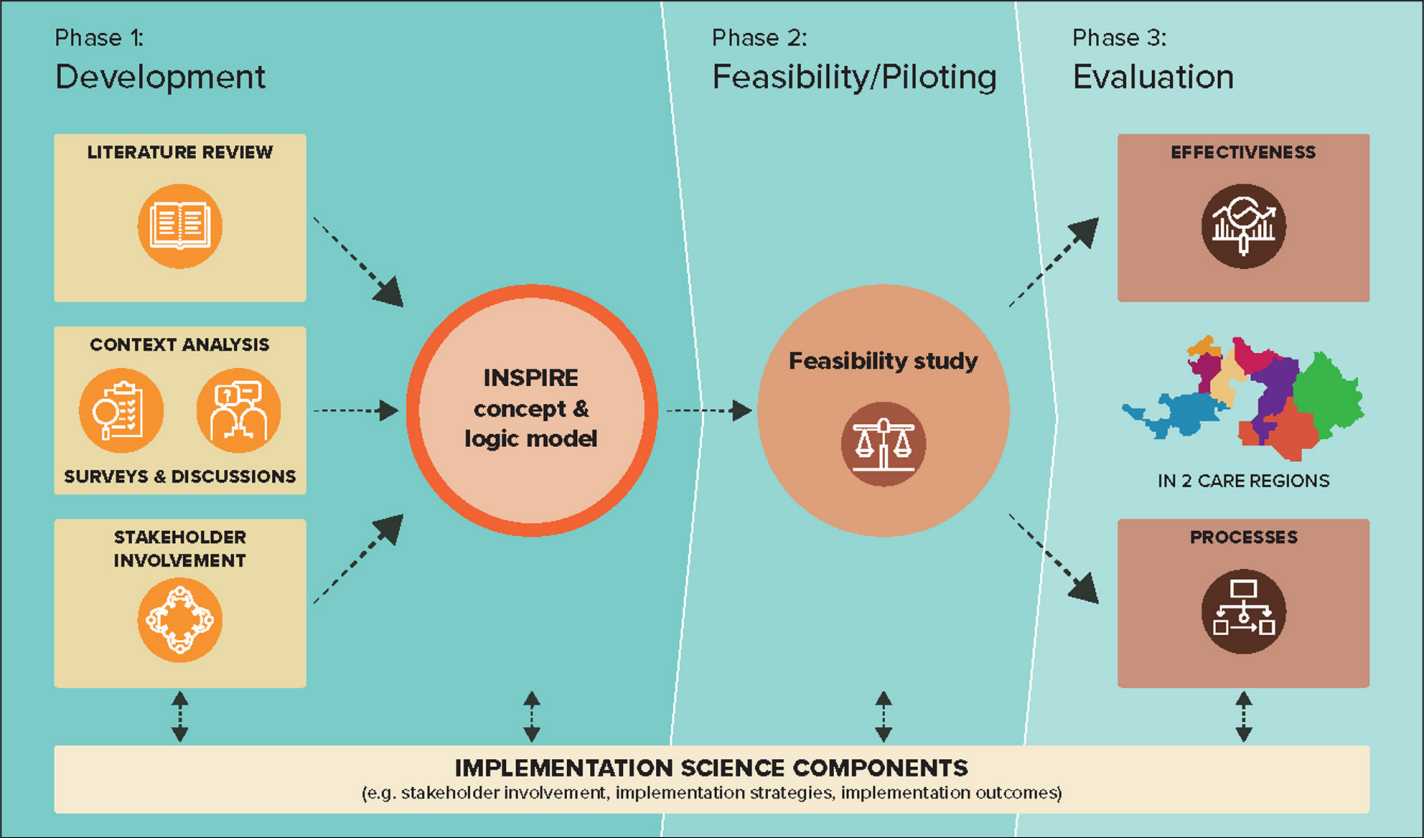
INSPIRE project overview mapped according to the Framework of the Medical Research Council.

The overall INSPIRE project is positioned within phases one to three of the Medical Research Council (MRC) framework for developing and evaluating complex interventions, yet also includes implementation science elements, such as a contextual analysis, stakeholder involvement, mapping of implementation strategies, and using a hybrid implementation-effectiveness evaluation (See ***Figure 1***). This paper specifically addresses the development phase of INSPIRE and aims to:

- Determine the contextual factors which may influence the INSPIRE integrated care model for the IACs and implementation strategies by collecting information through various sources
- Develop a logic model to display the overall theory for the INSPIRE care model, including inputs, activities, outputs, anticipated outcomes and assumptions
- Propose preliminary implementation strategies for the INSPIRE care model

**Figure 2:**
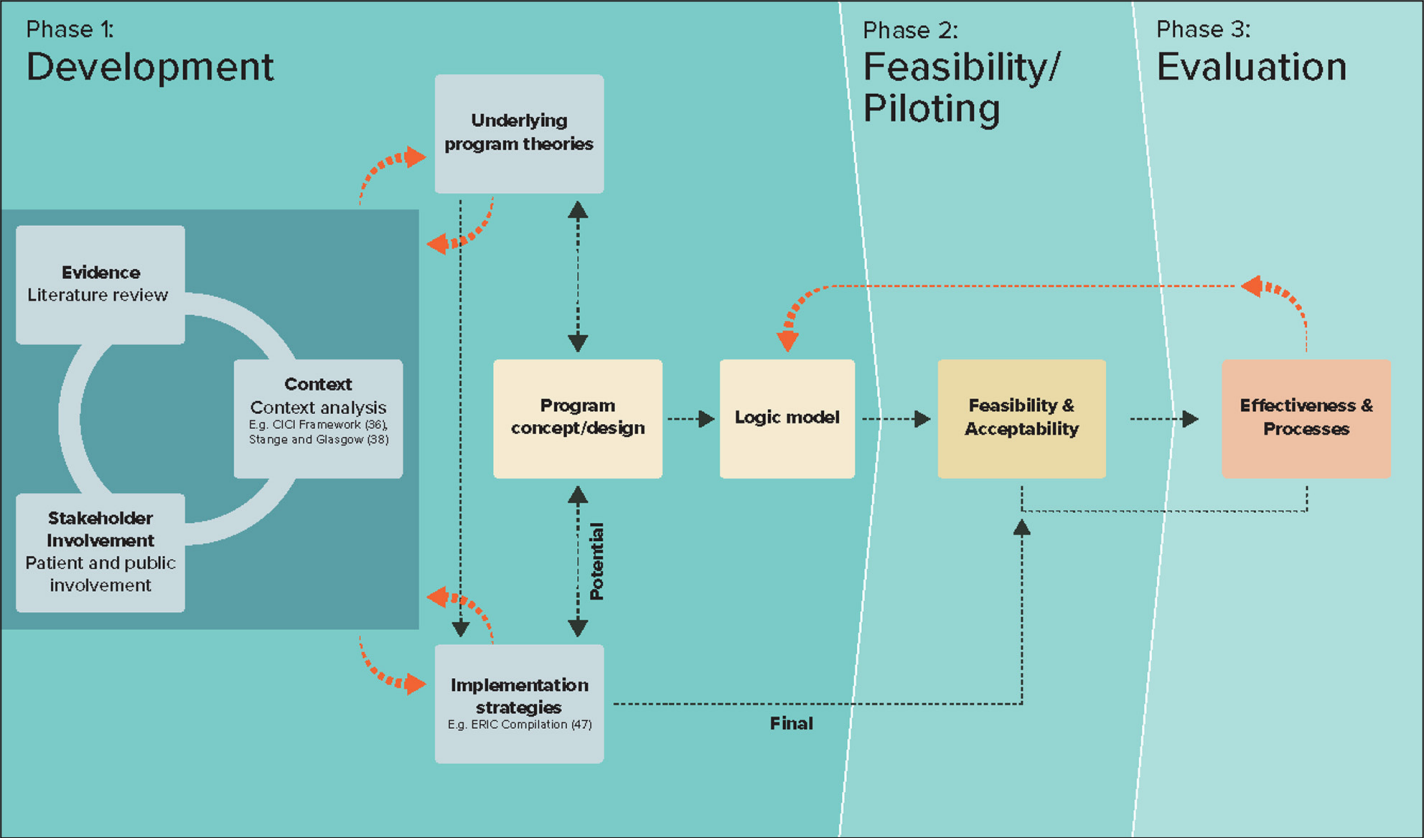
The INSPIRE project approach to care model development.

Performing the tasks related to these aims is a simultaneous and iterative process as shown in ***Figure 2***.

### Contextual analysis

We followed Stange and Glasgow’s (2013) approach to assessing context to identify, analyze and report on contextual factors which may influence the INSPIRE care model [38]. Their approach involves gathering contextual input from various stakeholders and using theories or frameworks to determine relevant ‘domains’ from which quantitative and qualitative information related to contextual factors should be collected, assessed, and reported [38]. Pfadenhauer’s CICI framework was used to identify which contextual domains to consider (e.g., political, socio-economic, socio-cultural), and how these contextual factors and the setting may interact with the INSPIRE intervention and its implementation [36]. We used a worked example in Pfadenhauer’s paper [36] as a template (Additional File 1) to synthesize our collected data related to the context, setting, and anticipated implementation. The data came from a combination of activities initiated by the research team, such as: conducting the INSPIRE cantonal stakeholder meetings, a cross-sectional survey and the context analysis meetings; participating in local stakeholder meetings; conducting the Basel-Landschaft Older Persons Survey [44]; and reviewing local, national and international reports (e.g., a key document by Threapleton et al. on implementation facilitators and barriers [14]) (***Table 1***). We mapped the identified contextual factors according to the CICI framework, and subsequently refined the INSPIRE care model components, the implementation process, and the potential implementation strategies.

**Table 1:** Data sources used for the contextual analysis.


DATA SOURCE	PARTICIPANTS INVOLVED	N	MODE OF DATA COLLECTION	PURPOSE OF DATA COLLECTION

INSPIRE Project Cantonal stakeholder meetings	The project team invited various individuals to the first INSPIRE Project Cantonal stakeholder meeting from organizations who are relevant in supporting or caring for the older population. New members are continuously welcomed, and participation has now grown to up to 70 members	5 cantonal stakeholder meetings organized by the INSPIRE research team between January 2018 and December 2019, with the number of participants ranging between 20-40 per individual meeting. Correspondence was sent via email from the INSPIRE account.	In-person meetings	To help the INSPIRE team stay informed on current happenings; identify relevant barriers and facilitators; inform stakeholders and discuss their input and concerns related to various project components (e.g., the care law “Altersbetreuungs- und Pflegegesetz”, the early prototype of the IAC care model, and the Basel-Landschaft Older Persons Survey).

Cross-sectional surveys and informal follow-up meetings to confirm interpretation of findings	A selection of representatives who participated in the first INSPIRE Project Cantonal stakeholder meeting	12 completed surveys	Electronic survey with 11 open-ended questions	To identify organizational interest in INSPIRE, current practices related to the intervention, perceived gaps in the health care supply, views on the role of the nurse in the IAC, as well as potential barriers and facilitators in implementing the IAC.

INSPIRE Project local stakeholder meetings	Health and social care providers and political representatives from specific care regions within Canton BL	Meetings are organized by the care regions approximately monthly (approximately 11 to date), and INSPIRE has been invited as a participant	In-person meetings	To discuss planning and implementation of the care model in local practice with the working groups from specific care regions.

Context analysis meetings	A local General Practitioner’s office, Specialist Centers for Ageing Issues and home-care providers in selected care regions	5 meetings were arranged by the INSPIRE research team	In-person meetings and semi-structured interviews	To get an overview of the daily processes and activities related to the care of older adults in each setting, and learn how to work together with current providers to support the IAC implementation.

Local, national and international papers and reports related to integrated care or caring for multi-morbid/frail older adults	n/a	n/a	Report/article review and data extraction	To increase research team’s awareness and understanding of the background, trends, and recent evidence, and inform the thinking about relevant factors to consider with respect to the integrated care model.

Basel-Landschaft Older Persons Survey	n = 8,786 valid questionnaires were completed by home-based adults aged 75 and older living in Canton BL	*More details reported elsewhere* [44]	Quantitative paper survey	To understand the living preferences of home-based older adults in Canton BL as well as the support and services they require and anticipating needing in future to make ageing in place possible.


BL = Basel-Landschaft; IAC = Information and Advice Center.

### Development and validation of the logic model

#### Development

A logic model describing the input/resources, activities, anticipated outcomes and impact was created to illustrate the overall INSPIRE program theory for how the IAC could function to achieve the desired results in the community. The template and definitions for each logic model component were based on the W.K. Kellogg Foundation [32], the Canadian Evaluation Society [45] and the Centers for Disease Control and Prevention [30]. The one-page logic model illustrates the outcomes chain (i.e., the successive relationship between the immediate, intermediate and long-term outcomes) in the program theory and some of the assumptions about “program factors” (e.g., effective advertising of the IAC), “nonprogram external factors” (e.g., participant factors that can potentially influence the outcomes) and the change process [31,32]. The logic model was built based on a deductive approach to constructing program theory as the ideas were gathered from documentation such as the data sources for the contextual analysis, grey and peer-reviewed literature, and program documents developed by the research team [31]. As logic model development is not a static process, it continued to evolve as we gathered contextual information, detected gaps in our program theory, and identified additional types of implementation strategies needed (e.g., *train and educate stakeholders* and *engage consumers*) [46,47].

#### Validation

The original core components of the INSPIRE integrated care model were presented during in-person cantonal stakeholder meetings to ensure that the overall model appeared to be appropriate from the perspective of local professionals. To gather stakeholders’ opinions on the program logic model, we undertook a structured activity during a stakeholder meeting attended by 40 stakeholders (e.g., health and social care organizations/providers, cantonal and municipal representatives, patient organizations, umbrella organization for care homes, volunteer organizations, health insurers, etc. [48]). We showed the stakeholders a condensed German version of the logic model that included the resources, activities, and outputs, but excluded outcomes. We asked stakeholders to work in groups to create a list of outcomes, i.e., the differences they expect to see as a result of the INSPIRE care model. Groups contributed their input via online interactive presentation software. Stakeholders were asked to choose from the long-list the three most relevant outcomes, resulting in a final list. Following the meeting, their input was incorporated into the INSPIRE logic model to create a new version, which was subsequently emailed to the stakeholder group for further input, and to identify any gaps or revisions needed.

### Deriving preliminary implementation strategies

Determining implementation strategies which fit the context is a two-step process involving an analysis of the factors which may influence implementation, followed by a selection and tailoring of implementation strategies [42]. In the current study, we mapped contextual data to the CICI framework, and synthesized this information to derive actions needed in terms of the care model and preliminary implementation strategies. We also reflected on the implications for the intervention or implementation strategies based on the contextual factors.

The implementation strategies were specified according to the Expert Recommendations for Implementing Change (ERIC) compilation [28,47,49], and were added to the logic model to indicate the actors and outcomes they intend to influence. This is a preliminary selection of strategies which will be systematically mapped, assessed for their evidence level and reviewed by stakeholders.

### Bringing it all together

As shown in ***Figure 2***, the INSPIRE project team used a unique approach to perform the preparatory work when designing the care model that aligns with O’Cathain’s recommendations [50]. As a first step, the project team performed a literature review, context analysis and involved stakeholders to develop the underlying program theory for how the intervention could work. As this is a circular process, specific details of the program theory and operationalization of the program progressed through stakeholder feedback or as more empirical data surfaced over time. Likewise, potential implementation strategies transpired as a result of the evidence, context and stakeholder input, as well as through the evolution of the program theory. The program theory was then formulated into a preliminary concept for the care model, accompanied by potential implementation strategies. To operationalize and communicate the program theory, a logic model was drafted and regularly adapted for one year. The final implementation strategies will evolve based on their success or failure, and as new information becomes available.

### Ethical considerations

This study was submitted to the Ethikkommission Nordwest- und Zentralschweiz (EKNZ) in Switzerland, EKNZ Project ID Req-2019-00900. The study was able to be conducted as the EKNZ deemed that it complied with the general ethical and scientific standards for research with humans (Art. 51 Abs. 2 HRA) and did not meet the definition as a research project requiring further review as per the Human Research Act ART.2.

## Results

### Context analysis

We selected specific contextual domains according to the CICI framework, including: socio-economic, socio-cultural, political, legal, epidemiological and the setting (Additional File 1). Key contextual factors on a macro level included: a lack of national integrated care regulations; the presence of integrated care guidance and indications of political support for integrated care; potentially challenging financing models; and inconsistent IT systems. Additionally, we noted the significant changes in nursing education across Switzerland over the past several decades, which is an important consideration when hiring an appropriate nurse for the IAC. On a meso level we noted: the rapidly growing population of older adults in BL; a cantonal law aiming to improve care for older adults yet not specifying the organization of integrated care; and the numerous organizations involved in the care of older adults. We also found that approximately one quarter of home-based older adults (aged 75+) in Canton BL showed signs of frailty, but that health care professionals likely do not systematically screen for frailty nor do general practitioners (GPs) typically perform a comprehensive geriatric assessment (CGA). On a micro level, we observed that the IAC and the nurse position would be new for the community and therefore new processes and tools, such as a referral pathway, an electronic patient file and communication tools, would be needed for the professionals to work together to deliver person-centered integrated care. In terms of the setting, the function of existing community-based centers that are mainly staffed by social service professionals and provide advice (e.g., social/financial) to older adults, could potentially be morphed into the new IACs required in the care law.

### Logic Model

**Figure 3:**
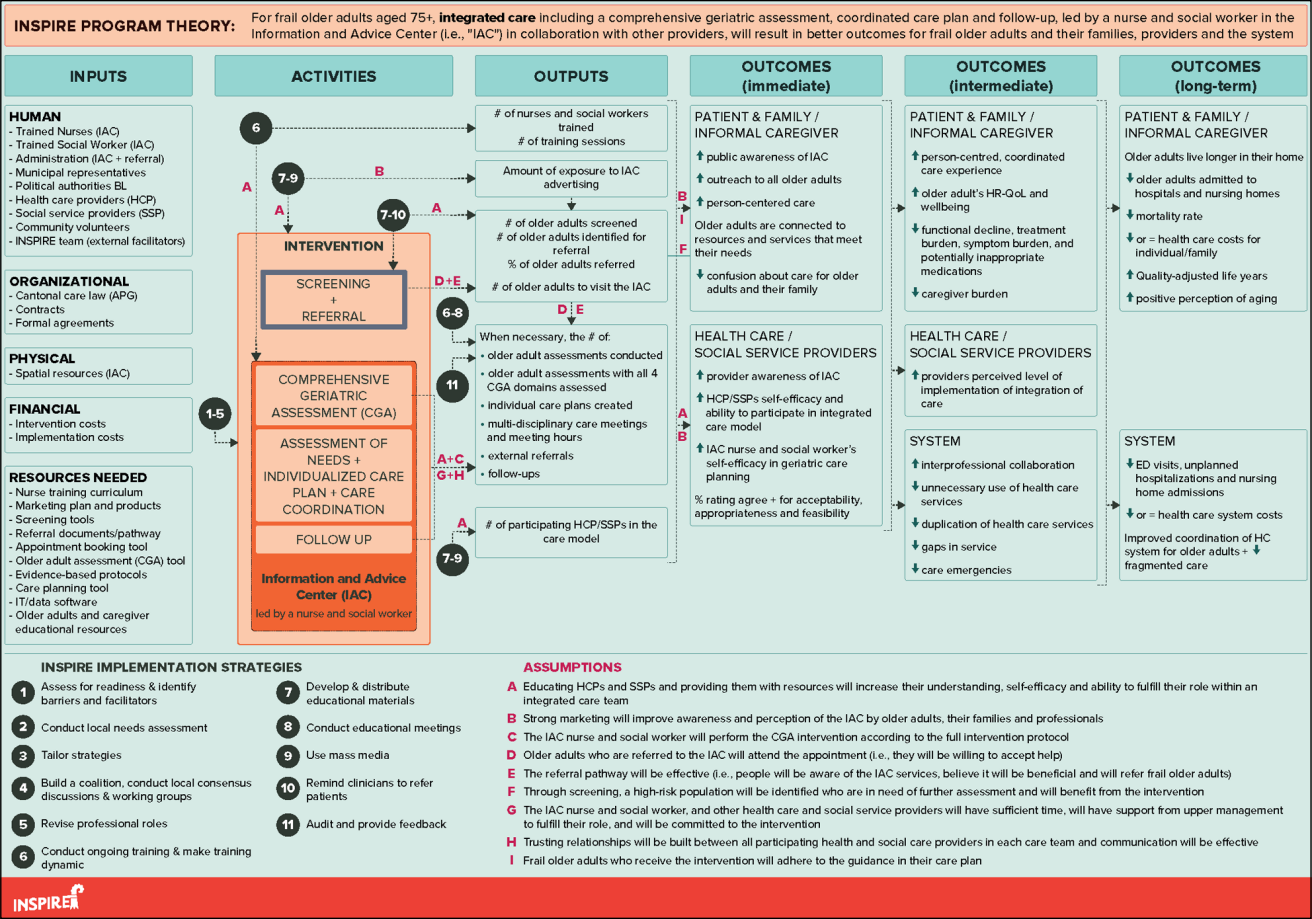
The logic model for the INSPIRE care model. BL = Basel-Landschaft; CGA = Comprehensive Geriatric Assessment; ED = Emergency Department; HC = Health care; HCP = Health care providers; HR-QoL = Health-related Quality of Life; IAC = Information and Advice Center; SSP = Social service providers.

The INSPIRE logic model illustrates the program theory for a care model that integrates health and social care service provision for home-based frail older adults (***Figure 3***).

The inputs column lists the resources that will contribute to the operation of the care model within any care region in BL. This covers human resources, such as the people referring older adults to the IAC, IAC employees, and stakeholders involved in decision making/funding, as well as organizational resources, such as the care law which mandates the IAC. It also includes the physical space where the IAC services will be delivered, the costs to run and support implementation of the IAC, and other resources (e.g., tools for screening and conducting a CGA, as well as marketing products).

The activities include the core components of the INSPIRE care model. First, individuals will be screened to identify those at risk of health deterioration who could benefit from in-depth geriatric assessment and coordination of additional services. Screening older adults aged 75+ for a certain geriatric risk profile indicating potential frailty can be performed by older adults, family members or health/social care professionals in the community such as GPs. At-risk older adults can be referred for an appointment at the IAC. The core components of the care model in the IAC, include conducting a CGA by a geriatric nurse expert and social worker; creating an individualized care plan including evidence-based interventions with a multidisciplinary team; and needs-based follow up. The geriatric nurse expert will act as the care coordinator in close collaboration with the social worker, and the care plan will be rolled out with the older adult and their caregivers, the GP, and health and social services in the community. The outputs column describes the main products anticipated as a result of both the intervention components and the implementation strategies. Certain aspects that will contribute to the measurement of implementation outcomes (e.g., acceptability, appropriateness, feasibility and fidelity) are reflected in the outputs and outcomes columns. For example, the percent of eligible older adults who receive an individualized care plan will contribute to the measurement of the intervention fidelity, and the IAC nurses’ views on whether the intervention is appropriate for frail older adults will be explored to measure intervention appropriateness. The outcomes columns are grouped temporally based on when we anticipate the change will be seen.

Lastly, arrows indicate the links between the activities and outcomes. These links illustrate the sequential outcomes anticipated and are evidence-informed based on clinical expertise, expert opinion, recommendations or previous/current hypotheses. To provide three examples: first, we anticipate that performing a CGA including a care plan and follow-up, which involves multi-disciplinary care professionals, will result in a care plan coordinated by one professional based on the older adults’ needs, being connected to necessary resources and services, and improving person-centered coordinated care. If the health and social needs of the older adults are assessed, we anticipate this will result in appropriate referrals, which may help to reduce the pressure on caregivers. As a second example, the educational meetings held will be instrumental to increase awareness of the IAC, and to determine how care planning can best be coordinated between the IAC and the other health and social service professionals, such as GPs. Thirdly, reviewing the patients’ medication list by the geriatric nurse expert as part of the CGA should help to flag any potentially inappropriate medications, which can be a concern with community-dwelling older adults.

During the validation phase, stakeholders elicited similar outcomes to those anticipated by the project team, and contributed new valuable outcomes. There were no concerns or discrepancies regarding the logic model when the revised version was sent to stakeholders.

### Implementation strategies

***Table 2*** presents the implementation strategies which were selected from six different ERIC clusters, namely: *Use evaluative and iterative strategies*; *adapt and tailor to context*; *develop stakeholder interrelationships*; *train and educate stakeholders*; *support clinicians*; and *engage consumers* [49]. For example, the strategy “use advisory boards and workgroups” was operationalized in our project by collaborating with local workgroups, including social service professionals to co-develop the electronic patient file which will be used for the IAC consultations. Meanwhile, given the diversity in the nursing education system in Switzerland, ongoing training is planned for the IAC nurse to increase their self-efficacy in geriatric care planning and to fulfill their role, as marked by the training-related strategies. In terms of educating stakeholders, it will be crucial to provide GPs as well as other providers in the community with information about the IAC, a referral path, as well as communication tools to foster care coordination. We anticipate that additional strategies will be needed as implementation progresses, depending on the resources available.

**Table 2:** Potential implementation strategies for INSPIRE presented using the Expert Recommendations for Implementing Change (ERIC) compilation [49].


ERIC CLUSTER	ERIC IMPLEMENTATION STRATEGY	Description of the implementation strategy in the INSPIRE project

**Use evaluative and iterative strategies**	Assess for readiness and identify barriers and facilitators	To identify barrier and facilitators, a contextual analysis was conducted. Readiness has been assessed through communication with cantonal stakeholders and local care regions.

	Audit and provide feedback	A form of auditing and feedback will be provided to the IAC nurse and social worker based on data collected in the feasibility study.

	Conduct local needs assessment	Local needs, gaps and current care processes were assessed through the contextual analysis. To gather a deeper understanding of older adults’ experiences of their health and social needs and care, a population survey was conducted with older adults in Canton BL and interviews are planned with frail older adults.

**Adapt and tailor to context**	Tailor strategies	Potential implementation strategies have been and will continue to be selected based on the contextual analysis, stakeholder input, and strength of evidence. New strategies will be selected during the feasibility phase based on any emerging barriers and discussed with stakeholders.

**Develop stakeholder interrelationships**	Build a coalition	The INSPIRE team established a Cantonal stakeholder group that aims to meet quarterly to discuss matters related to the IAC, the care model, the INSPIRE project activities and the new care law. The INSPIRE team also collaborates on a local level with workgroups. Local GPs will be engaged separately as an important stakeholder.

	Conduct local consensus discussions	

	Use advisory boards and workgroups	The INSPIRE team participates in any working groups (which include local politicians and frontline social service professionals) that focus on implementation of the IAC within a selection of care regions, where the care model components and study design are discussed. The social service professionals and the research team are co-developing the electronic patient file for the IAC, which is the tool that will be used for consultations with older adults.

**Train and educate stakeholders**	Conduct ongoing training	A training curriculum has been co-developed for the IAC nurse(s) which includes input from the contextual analysis. In-person and online training modules are planned for the IAC nurse(s). If needed, training or education sessions will be planned for the IAC social workers.

	Make training dynamic	

	Develop educational materials	Educational materials will be co-developed with care regions and distributed to inform health and social service providers about the new IAC services, how to screen and refer at-risk older adults to the IAC, and the goals and process of integrated care planning. Any changes to the IAC services would also be communicated to these local professionals.

	Distribute educational materials	

	Conduct educational meetings	An event with local GPs is being planned in collaboration with stakeholders which will include discussion of the IAC. Additionally, a marketing plan and educational session will be planned with stakeholders for community providers to learn about the IAC, including their roles as community professionals, and provide their feedback.

**Support clinicians**	Remind clinicians	A mechanism could be suggested to remind GPs and home care nurses to screen older adults with a certain geriatric risk profile, and refer them to the IAC if appropriate.

	Revise professional roles	Roles and responsibilities will need to be clear for the IAC staff, and emphasizing the goals of care continuity and coordination between professionals. The INSPIRE team co-developed a job description for an IAC nurse and emphasized the importance of the role of the social worker in continuously collaborating with the IAC nurse to effectively co-deliver integrated care services. An integrated care pathway will be created to clearly outline the different roles of the professionals. The electronic patient file for the IAC may also help to delineate the roles of each professional.

**Engage consumers**	Use mass media	Advertising materials should be co-developed with care regions to inform older adults, their family, care professionals and community members about the new IAC services. Consistency in advertising will be important.


BL = Basel-Landschaft; IAC = Information and Advice Center; GP = General Practitioner.

## Discussion

Given the international desire to establish effective models of integrated care for home-based, frail older adults, this paper described the essential investments made during the development phase before implementing a new integrated care model in Canton BL. The results of this study demonstrate how a rich understanding of the context can help further refine an intervention concept and consider preliminary implementation strategies. Additionally, a contextually-relevant logic model was created to effectively communicate the program theory to INSPIRE project members, stakeholders, and other researchers.

Overall, many of the activities, outputs and outcomes described in our logic model are comparable with those seen in the Social Care Institute for Excellence Logic Model for Integrated Care [51] and the Logic Model for Patient-Centered Medical Home Models [52], among others [53,54]. Nevertheless, our logic model is specifically designed for our program and context, incorporates our assumptions, has an operational-level focus, and includes our implementation strategies. By providing a rich description of the contextual factors collected to date, this study addresses a common gap in the literature where the context of interventions is often not reported or only vaguely described. Without the findings emerging from the contextual analysis, necessary actions related to the intervention or implementation strategies would not have been detected. Examples of this include: the future role of IACs in performing a CGA and care coordination based on the current care system; identifying the local health and social service organizations to coordinate care with and to prevent duplication of services; the importance of a marketing plan for the IAC and unique strategies needed to reach family members; and the competencies needed by the IAC nurse and how defined pathways could help them work together with the social worker and GPs. Additionally, the importance of early involvement of professionals in the community, such as GPs, to facilitate frailty screening and referral to the IAC and collaborative care planning. However, some of the contextual barriers will remain outside of our control within the project, such as the financing models, incentives for integration or whether electronic records are shared across the whole system [14]. Awareness of these factors will also allow for a more accurate evaluation of the care model in future and interpretation of the results, and can also support other researchers or professionals who are looking for guidance on how to analyze context and use the findings within their intervention development. Stadnick et al. (2019) recently conducted seven case studies of integrated care initiatives across multiple countries, where they reflected on the shared contextual factors which influenced the implementation of these projects [55]. Among the inner context factors, several of the important considerations identified such as knowledge, education, training and confidence of service providers; monitoring fidelity; and shaping providers’ roles and responsibilities, will be relevant in the INSPIRE project and can guide where to enhance our efforts [55]. Establishing a “community-academic partnership” was the main bridging factor they identified [55], which will remain of great importance during all phases of the INSPIRE care model. By describing and linking the ultimate program goals with the activities that will be done to achieve these goals, the logic model revealed our thinking about what should work and how [31,32,33], mitigating the “black box” phenomenon which can otherwise occur when describing an intervention [46,52].

With respect to the overall program theory, the integration of health and social care has been fundamentally endorsed for years [13,56,57,58], especially for populations with complex needs [59,60]. The program theory encompasses the World Health Organization’s approach to *Integrated Care for Older People* (ICOPE) at the micro-level [60], and at the meso-level it incorporates actions deemed essential based on findings from the recent eDelphi study on implementing the ICOPE approach (e.g., conducting comprehensive assessments and training personnel to develop a care plan) [61]. As the first component in the care model, screening for potential frailty has been promoted as part of a preventative approach and as an effective means to determine the subset of the older population that would benefit from further comprehensive assessment and subsequent interventions [62,63,64,65]. Given that only a subset of the older population is estimated to be in higher need of IAC services, screening for potential frailty is particularly appropriate to use healthcare resources efficiently, combined with the recognition that frailty is an emerging public health priority [66]; and that it is likely a major factor predicting admittance to a nursing home [3]. Although there are different schools of thought on whether and how to screen older people for frailty in different health care settings based on feasibility, evidence gaps and resources required [65,66,67,68,69], frailty detection is essential to determine actions which can help prevent further conditions associated with aging [66]. If supported by appropriate implementation strategies, we believe screening can be an effective mechanism for identifying older adults most in need of further assessment.

Following screening, the remaining activities included in the program theory (i.e., conducting a CGA; assessing needs; creating and coordinating a care plan; and conducting follow-up) are highlighted as part of an integrated care approach for frailty or multi-morbidity [11,13,14,15,70,71,72], and are common to many studies of this nature [19,65,73,74,75,76]. The core intervention features a CGA at the center, which is considered either beneficial or a gold standard in caring for frail older adults in certain settings [66,77,78,79]. In a recent scoping review of 27 integrated care programs for older people, the authors found that the 21 different CGA instruments used incorporated three of the dominant principles of integrated care, i.e., comprehensive, multidisciplinary and person-centred care [80]. However, they proposed that stronger involvement of both social care professionals and older adults could strengthen the CGA process, which will be key in the INSPIRE model [12]. The present study, together with results from the ongoing systematic review by Briggs et al. assessing the effectiveness of the CGA in community-dwelling, frail older adults [81], will help add to the body of research testing the CGA as part of an integrated care model to improve outcomes for this population.

The program outcomes presented in the logic model were derived from studies of related care models [9,10,11,82,83,84,85]; outcomes that have been proposed for integrated care initiatives [8,86]; the program team’s realistic assumptions and/or stakeholder expectations (e.g., relief and support, coordination, costs and perception of aging). Achievement of these outcomes relies on important assumptions such as trusting relationships and strong communication between providers [87] as well as “provider commitment to and understanding of the model” [88]. However, previous authors have questioned whether some of the outcomes hypothesized for integrated care for this population are in fact appropriate or realistic, such as improvements in activities of daily living or quality adjusted life years [9,10]. Focusing on care processes and outcomes that are most important to patients have been emphasized as a priority [9,10,85], particularly measuring patient’s care experience as an outcome to reflect the quality of integrated care for multi-morbid individuals [89] or concentrating on intrinsic capacity and the patient’s individual goals [59].

With respect to the process of intervention development, O’Cathain et al. (2019) conducted a consensus exercise with experts to offer guidance for intervention development [50]. We endorse the principles they put forward, as we illustrated a “dynamic, iterative, creative, open to change and forward looking” approach in our process [50, p.2]. Our paper provides a practical example of how some of the actions within their framework (e.g., “involving stakeholders, reviewing published research evidence, drawing on existing theories, articulating programme theory, undertaking primary data collection, and understanding context”) can be applied and combined to prepare for a new intervention. It also reflects on the relationship between these steps with emphasis on logic model development and adds the element of implementation strategies. While the process and results are specific to our project, the approach and methods we used for the development phase can be broadly generalizable for other researchers, which is a strength of this study.

### Methodological Considerations

With regards to contextual analysis, new methods are under development to guide researchers in the field to use a consistent, systematic approach for analyzing context [90]. As context constantly evolves, the factors present at the time of data collection may change and therefore it will be important that the implementation strategies adapt along with it. Contextual factors will differ for every setting limiting the generalizability of the care model; therefore, we have made the contextual factors in our situation transparent for researchers. In essence, the overall methodological approach can guide researchers for assessing their own setting, designing a logic model, and to facilitate the design and evaluation of future care models.

Logic models can also be criticized for not describing “why” activities produce outcomes that would otherwise be clear through a theory of change [91]. Another downfall is that some more basic or linear versions may fail to capture context or communicate the true complexity involved for a complex intervention to become contextually-fit [92,93]. However, our format supports program planning and was appropriate for our purposes, as described by Mills et al. [92]. Nevertheless, the combination of the logic model, extensive narrative describing the evidence-informed strategies, and contextual analysis we provided in compliment should support interpretation of the logic model and understanding of the development of this complex intervention [36]. As an alternative means, other authors have described innovative methods they used to account for context while developing logic models [46,93], and have proposed a new format for presenting this [92]. Innovative work by Smith et al. (2020) may support implementation science researchers moving forward as they have introduced new templates for logic models that link the different frameworks specific to implementation science and can support the various study designs [94]. For the future evaluation of the care model, a systems thinking approach may be more appropriate to accurately reflect the complexity of the system [95].

## Conclusion

This study has set the foundation for the next steps in the INSPIRE research project: to conduct a feasibility study of the integrated care model and implementation strategies prior to full evaluation of the implementation and intervention outcomes. Based on the insights of previous integrated care studies on older adults, stronger understanding of context and program theory is needed, especially to develop, implement and evaluate these initiatives which are yet to yield strong evidence in the field. Investing sufficient efforts into program development and stakeholder involvement is essential to ensure a strong fit between the context and the integrated care model, identify the implementation strategies needed, and reduce research waste. Flexibility in the next phases of research and implementation will also be essential as changes in leadership, policies, and so on is typically inevitable. The approach followed during this study can be used as a basis and adapted when developing future integrated care programs.

## Data Accessibility statement

The datasets used and/or analysed during the current study are mostly included in this published article and the sources are listed in ***Table 1*** or Additional File 1. Other data files can be requested, provided the identity of the data sources will be kept anonymous.

## Additional File

The additional file for this article can be found as follows:

10.5334/ijic.5607.s1 Additional File 1. Applying the Context and Implementation of Complex Interventions (CICI) framework to the INSPIRE project.
