# Supplementary material for: A Contextual Analysis and Logic Model for Integrated Care for Frail Older Adults Living at Home: The INSPIRE Project
Source: Int J Integr Care. 2021 Apr 23;21(2):9. doi: 10.5334/ijic.5607 (PMC8064293; doi:10.5334/ijic.5607)
Supplement: Additional File 1. — Applying the Context and Implementation of Complex Interventions (CICI) framework to the INSPIRE project. [file ijic-21-2-5607-s1.pdf]

Additional File 1: Applying the Context and Implementation of Complex Interventions (CICI) framework to the INSPIRE project

SETTING	Description of the setting for a new integrated care model (a complex intervention)	Implications for the INSPIRE intervention
**Setting**	**Macro**<br>• Switzerland<br>**Meso**<br>• Canton Basel-Landschaft (BL) in the German-speaking part of Switzerland<br>• Municipalities, organizations and professionals within Canton BL are affected by the Cantonal care law requirements (APG) to form new care regions and establish an information and advice center (IAC) for older adults [1]<br>• Numerous organizations are involved in providing services or support for home-based older adults in Canton Basel-Landschaft, such as: 30+ different home care organizations, patient organization for older people, an umbrella organization for care homes, health insurance companies, meal delivery services, churches, transportation services, disease-related support services<br>**Micro**<br>• There are current Specialist Centers for Ageing Issues in some of the care regions which is a social service that older adults and/or their families can visit when they have social or financial concerns (however no medical advice is provided there)<br>• The work environment of the IAC will influence the implementation of the care model	• The new information and advice center (IAC), which based on the INSPIRE model will be an integrated care site combining health and social services, could potentially be embedded in the existing Specialist Center for Ageing Issues in certain care regions<br>• Co-location of the IAC nurse and social service provider may increase frequency and quality of communication and improve access to the appropriate professional knowledge<br>• Coordination with the other existing community services will be important
**CONTEXT**		
**Contextual domains and description**	**Description of the context for a new integrated care model**	**Implications for the INSPIRE intervention or implementation strategies**
**Socio-economic context**	**Macro**<br>• Funding for health care in Switzerland comes from multiple private and public sources, including high out-of-pocket expenses that are covered by patients/individuals [2]<br>• Funding for the social security system and social support services come from public and private/semi-private sources<br>• Basic health insurance in Switzerland is mandatory, with the option to purchase supplementary health insurance [2]<br>• The financing models, rate structures, and limited cost incentives for health care integration have been recognized by Swiss Cantonal representatives as very significant obstacles to providing integrated care in Switzerland, and requiring need for action [3]	• The financing models in Switzerland may present challenges for coordinating care with providers who will be involved in the care plan of an older adult who visits the IAC<br>• The IAC care model and services may be faced with uncertainty, lack of clarity from professionals and implementation delays due to its position within a larger care law<br>• There are important funding and service gaps for the IAC staff to be aware of

<table>
<tr><td></td><td>
The compensation systems in Switzerland are based on individual institutions or professional groups, which make networking and coordination a challenge [3]
Meso
Local stakeholders shared concerns regarding the ability to coordinate/synchronize the different health and social services when organizations have different funding, requirements, and mandates, and the APG does not set explicit details regarding funding
Local stakeholders have recognized funding gaps for individual preventative services, programs for certain populations and for care and support at home
Micro
An international survey of adults aged 65+ (n = 1,084 in Switzerland) found that 22% of adults aged 65 or over spent 2,000 dollars or more out-of-pocket in the past year on care [4] (however, this must be interpreted within the Swiss context)
</td><td></td></tr>
<tr><td>Socio-cultural context</td><td>
Macro
A survey across Switzerland in 2015-2016 identified an upward trend in integrated care initiatives. As part of the innovative research in health care services, coordinated care is seen as one priority evidenced by funding for coordinated care initiatives [2, 5]
Swiss Cantonal representatives have recognized the conflicting interests of all actors involved as a very significant obstacle to providing integrated care in Switzerland and requiring need for action [3]
Often family members of older adults provide support instead of, or in addition to, using formal long-term care services, partially due to the costs [6, 7]
There have been many changes in nursing education across Switzerland over the past several decades, therefore there is a mix between nurses who were trained in for example higher education programs, colleges, or hospital-based nursing schools, as well as other vocational training [8]
Differences can be expected between the health and social sectors in terms of accountability, professional backgrounds, values, instruments used and so forth
Meso
There are already multiple centers and services for older adults, many of which may be well-utilized
Many local health and social care organizations report actively working with each other, however also perceive a lack of coordination and collaboration between some of the services, organizations and service providers in the Canton
Examples of gaps reported by local health and social care organizations include: limited relief services for caring relatives or affordable intermediary service, limited care or engagement with the subgroup of older adults that could be very old, lonely, less mobile or don’t have support from family or financially, gaps in outpatient service for 24 hours of care, lack of coordinated services, lack of concrete case management and insufficient night coverage services
There is insufficient information on services available (including for caring relatives) and also
</td><td>
It is important that the IAC services do not overlap with the existing services offered in some of the care regions to avoid duplication. IAC staff should be aware of these services to ensure optimization
The services delivered at the IAC should strive to: improve coordination between professionals in the canton, provide a comprehensive list of all the offers available for older adults and caring relatives (e.g., health, social and housing offers and services, including all of the different home care organizations and costs involved), provide case management when necessary, have strong awareness of the situation and gaps related to current services in the care supply chain
Marketing of the IAC will need to be well planned
Communication/support is needed for family members of older adults who would benefit from IAC services
Unique strategies are required to reach out to and approach isolated older adults who would benefit from care but do not demand it
When developing the materials and resources
</td></tr>
</table>

	contradictory information, therefore many older adults, their family members, and community professionals may not be aware of the full range of services available<br>• There may be too many contact points available for people when needing support, instead of one central contact point<br>• The transition to nursing homes could be improved<br>• Primary and community health and social care providers are not often brought together to discuss the needs of individual older people [9]<br>• Communication between professionals varies between formats depending on who the communication is with and GPs often do not meet with other providers<br>• There may be many older adults who choose not to ask for help or are reluctant to using services available as they would prefer not to be a burden for others or highly value independence<br>• Canton BL has urban and rural parts, where different values and interests may be noted<br>• To avoid duplication (e.g., parallel structures offering the same consultations/clarification), the IAC needs to be perceived by the population as professionally competent and broadly based and ensure good coordination with the numerous existing centers<br>• There may often not be time for primary care GPs to conduct a comprehensive geriatric assessment of their patients	for the IAC, there are local organizations who can provide resources and knowledge<br>• Communication between GPs and the IAC staff regarding referral and care planning will need to be piloted<br>• IAC staff need to have close exchange and involvement with other providers. A new process flow as well as tools will be needed for the IAC staff to communicate and coordinate care with the older adult's other health and social professionals<br>• Many important topics will need to be included in the IAC nurse training depending on their level of knowledge and experience<br>• Home visits may be necessary to reach more frail individuals<br>• The INSPIRE project team should be aware of the different values when working with local professionals and older adults, as well as understanding the cultural change that is introduced by establishing the new care regions, for example
**Political and legal context**	**Macro:**<br>• Switzerland's health system has a federalist structure, and the responsibilities are divided at three levels: federal, cantonal and local. The system is decentralized and the 26 Cantons in Switzerland have a major responsibility for healthcare, and the local municipalities have a major role in long-term care [2, 10]<br>• Responsibilities within the social security system (e.g., old age pensions) are divided across the three government levels. Other social support services are often run privately/semi-privately<br>• Switzerland has no federal regulatory framework for integrated care [2]<br>• The Swiss Conference of Cantonal Health Directors (GDK) has published guidelines on integrated care for the cantons [3]<br>• Based on a recent survey, there appears to be political support for state actors to become more involved in integrated health care [3]<br>• Swiss Cantonal representatives demonstrated commitment in a recent survey to the integrated care goals related to improving cooperation between different professionals and organisations as well as	• Despite the absence of a federal or cantonal framework for integrated care, there are guidelines and appears to be political support for elements of integrated care, however there may be some challenges<br>• The APG mandates will require the municipalities to comply and organizations and professionals may therefore be motivated to cooperate in making the IAC successful for their community. However, guidance on integrated care is not specifically included in the care law, and a care model and new tools will be required for operationalizing the service.<br>• The political timelines will influence the

	coordination and continuity of care, however lower ratings to connecting the health and social systems [3]<br>• High diversity of IT systems in the Swiss health system and in companies that provide electronic health records. The use of an electronic health record for patients is mandatory for certain sites (e.g., hospitals and nursing homes), however it is voluntary for other healthcare providers including GPs [11]. Therefore, there lacks a nation-wide electronic health record system.<br>**Meso:**<br>• The APG in Canton Basel-Landschaft was introduced in January 2018 to improve care for the aging population and requires implementation of a new information and advice center (IAC) in each care region where needs are assessed and information and advice is provided to older adults, especially before entering a nursing home. The law specifically states that a nurse must be involved, that health promotion and prevention could be included in the IAC tasks, and that the center should be run independently to prevent a conflict of interest. The care law provides a high-level framework but does not provide detailed operational advice on how to carry out the guidance. The care law also does not provide guidance or common governance on how to deliver integrated care for older people in BL [1]<br>• Diverse IT systems for electronic health records<br>**Micro:**<br>• There are many political players and processes involved in forming the care regions and carrying out the guidance in the new care law<br>• Safe data sharing agreements or consent procedures will be required between different professionals involved<br>• The current Specialist Centers for Ageing Issues are not using a standardized electronic patient file that can incorporate documentation from multi-disciplinary professionals	intervention timelines<br>• The IAC should include health promotion and a preventative focus<br>• The IAC should aim to use an IT system that can cooperate with the various other IT systems used by the professionals in the Canton to improve care referral and coordination<br>• Explore opportunities to promote safe data sharing and electronic platforms for the IAC
**Epidemiological context**	**Macro:**<br>• In Switzerland (2018): proportion of people ≥65 was 18.5% and ≥80 years was approximately 5% [12]<br>• Switzerland has one of the highest life expectancy rates at birth [6, 13]<br>• In 2014, an international survey (n in Switzerland = 1084) found that 44% of adults aged 65 or older living in Switzerland were living with 2+ chronic conditions. 29% of adults aged 65 or older had experienced a coordination problem in the past two years. Of patients that had a chronic condition, only 47% had a treatment plan for their condition they could carry out in their daily life. Only 9% had a health care professional that between Doctor's visits contacts them to check in and only 58% said they can contact one to ask questions or get advice [4]<br>• In the next 30 years, there is forecasted to be an increased demand in long-term care in Switzerland [6]<br>• Based on international estimates, the majority of people prefer to live in their own home yet one-third of home-dwelling older people are at risk for not being able to age in place due to functional limitations [14, 15]	• The rapidly aging population and proportion of those living with multi-morbidities or frailty justifies development of innovative care models<br>• A screening tool will be needed for GPs and professionals to screen the older population and identify those in need of further health and social assessment at the IAC. A referral pathway will also be needed for the IAC<br>• The IAC should aim to support older adults to continue living at home and overcoming functional limitations<br>• The IAC should aim to improve coordination between professionals

	**Meso:**<br>• In Canton Basel-Landschaft (2018): proportion of people ≥65 years was 21.9% and ≥80 years was 6.4% [12]<br>• In Canton Basel-Landschaft: compared to 2018, population projection for adults aged ≥80 will increase by 27% in 2025 and 77,3% in 2040 [16]<br>• GPs in BL do not currently use a standardized tool to screen their elderly patients for frailty<br>• The INSPIRE Basel-Landschaft Older Persons Survey (conducted in 2019) of home-based older adults aged 75+ results identified that [17]:<br>○ respondents would prefer to live in their own home, even if they were to become more dependent<br>○ 1 in 4 respondents showed signs of frailty<br>○ respondents assumed in the future they may rely more on family members and organisations<br>○ note: additional results are reported in Deschodt et al (2020)	• The IAC staff could create a treatment plan and potentially be a contact for older adults between their other appointments

References

1. Altersbetreuungs- und Pflegegesetz (APG), in SGS 941. 2018. [Elderly Care and Nursing Law]. Available from: http://bl.clex.ch/app/de/texts_of_law/941/versions/2126. [in German].

2. Schusselé Filliettaz S, Berchtold P, Kohler D, Peytremann-Bridevaux I. Integrated care in Switzerland: Results from the first nationwide survey. Health Policy, 2018;122(6):568-76. doi:10.1016/j.healthpol.2018.03.006

3. Schweizerische Konferenz der kantonalen Gesundheitsdirektorinnen und -direktoren GDK. Impulse für die Integrierte Versorgung in den Kantonen: ein Leitfaden. Schweizerische Konferenz der kantonalen Gesundheitsdirektorinnen und -direktoren GDK; 2019. [cited 1 Jul 2019]. Available from: https://www.fmc.ch/_Resources/Persistent/710ed198d88654269f53c48c4697c0fbb08bda98/GDK_Leitfaden.pdf.

4. Osborn R, Moulds D. The Commonwealth Fund 2014 International Health Policy Survey of Older Adults in Eleven Countries [Powerpoint]. The Commonwealth Fund. 2014. [cited 3 Nov 2019]. Available from: https://www.commonwealthfund.org/publications/surveys/2014/nov/2014-commonwealth-fund-international-health-policy-survey-older.

5. Swiss National Science Foundation: NRP 74 Smarter Health Care: National Research Programme. [webpage on the internet]. [cited 8 Jan 2020]; 2020. Available from: http://www.nfp74.ch/en

6. Fuino M, Wagner J. Old-age care prevalence in Switzerland: drivers and future development. European Actuarial Journal, 2018;8(2):321-62. doi:10.1007/s13385-018-0185-3

7. Bonsang E. Does informal care from children to their elderly parents substitute for formal care in Europe? Journal of Health Economics, 2009;28(1):143-54. doi:10.1016/j.jhealeco.2008.09.002
8. Schwendimann R, Fierz K, Spichiger E, Marcus B, De Geest S. A master of nursing science curriculum revision for the 21st century – a progress report. BMC Medical Education, 2019;19(1):135. doi:10.1186/s12909-019-1588-9
9. Goodwin N, Smith J. The evidence base for integrated care. The King's Fund and the Nuffield Trust; 2011. [cited 3 Nov 2018]. Available from: https://www.kingsfund.org.uk/sites/default/files/Evidence-base-integrated-care2.pdf
10. De Pietro C, Camenzind P, Sturny I, Crivelli L, Edwards-Garavoglia S, Spranger A, et al. Switzerland: Health system review. European Observatory on Health Systems and Policies; 2015. [cited 14 Jun 2020]. Available from: https://www.euro.who.int/__data/assets/pdf_file/0010/293689/Switzerland-HiT.pdf
11. De Pietro C, Francetic I. E-health in Switzerland: The laborious adoption of the federal law on electronic health records (EHR) and health information exchange (HIE) networks. Health Policy, 2018;122(2):69-74. doi: https://doi.org/10.1016/j.healthpol.2017.11.005
12. Bundesamt für Statistik, Schweizerische Eidgenossenschaft: Interactive Statistical Atlas of Switzerland [Internet]. [cited 1 Jul 2019]. 2017. Available from: https://www.bfs.admin.ch/bfs/en/home/statistics/regional-statistics/atlases/interactive-statistical-atlas-switzerland.html [in German].
13. OECD. Health at a glance. Paris: OECD Publishing; 2015.
14. Wiles JL, Leibing A, Guberman N, Reeve J, Allen RE. The meaning of "aging in place" to older people. Gerontologist, 2012;52(3):357-66. doi:10.1093/geront/gnr098
15. Fuller-Thomson E, Yu B, Nuru-Jeter A, Guralnik JM, Minkler M. Basic ADL disability and functional limitation rates among older AMERICANS from 2000-2005: the end of the decline? The Journals of Gerontology: Series A, Biological Sciences and Medical Sciences, 2009;64(12):1333-6. doi:10.1093/gerona/glp130
16. Statistisches Amt des Kantons Basel-Landschaft: Kantonale Bevölkerungsstatistik, Altersprognose BL 2020 (Basis 2018). [cited 24 Jul 20]. 2018. Available from: https://www.statistik.bl.ch/web_portal/1_10_2_1
17. Deschodt M, Siqeca F, Yip O, Stenz S, Blozik E, Briel M, et al. Bevölkerungsbefragung Basel-Landschaft Kantonaler Bericht. Departement Public Health, Universitat Basel; 2020. [cited 20 Jul 2020]. Available from: https://inspire-bl.unibas.ch/wp-content/uploads/2020/07/INSPIRE-Kantonaler-Bericht-DE-200721-FINAL.pdf. [in German].
